# Optimizing the Method of Cell Separation from Bile of Patients with Cholangiocarcinoma for Flow Cytometry

**DOI:** 10.1155/2019/5436961

**Published:** 2019-05-02

**Authors:** Yafei Xia, Yuan Gao, Botao Wang, Hui Zhang, Qi Zhang

**Affiliations:** ^1^Department of Pharmacology, Tianjin Nankai Hospital, Tianjin 300100, China; ^2^Department of Oncology Surgery, Tianjin Nankai Hospital, Tianjin 300100, China; ^3^Tianjin Key Laboratory of Acute Abdomen Disease Associated Organ Injury and ITCWM Repair, Institute of Acute Abdominal Diseases, Tianjin Nankai Hospital, Tianjin 300100, China

## Abstract

Analysis of the change of the cells in bile is an evolving research field in biliary pathophysiology and has potential value in diagnosis and therapy. However, laboratory studies of cell in bile across the world are scarce. Bile was collected from the clinical patients with cholangiocarcinoma (CC). To optimize the cell separation method in bile of patients with CC, we studied the factors that may affect cell vitality in bile including the dilution buffer, centrifugal force, centrifugal time, and store time and temperature. Then these factors were modified and performance was evaluated by flow cytometry with respect to the percentage and total yield of viable cells. The separated cells from bile were stained with CD3, CD4, CD8, CD56, TCR*γ*/*δ*, CD16, CD14, HLA-DR, CD33, CD15, CD11b, lineage cocktail (CD3, CD14, CD19, CD20, and CD56), CD66b, and CD45 antibodies. The different buffer solutions were joined in bile of patients with CC; experiment results show that the different dilutions have nearly no effect on the ratio of cells in bile by flow cytometry. The best centrifugal procedure was 300 g, 10 min. Bile should be stored at 4°C rather than at normal temperature. Our study further showed that the shorter time of the bile storage, the higher viability of the cell, and immune cells existed in cells isolated from bile. Evaluating bile cell viability is necessary to evaluate method performance.

## 1. Introduction

Cholangiocarcinoma (CC) is a primary hepatic malignancy that arises along the intrahepatic and extrahepatic bile ducts [1–3]. CC, broadly described as a malignancy that arises from the transformation of cholangiocytes of the epithelial bile ducts, is an enigmatic and challenging cancer [4, 5]. CC is usually a fatal cancer that is extremely aggressive [6]. Although global morbidity and mortality are increasing, knowledge of the disease remains limited [7]. CC presents significant diagnostic challenges owing to its silent clinical character, the low specificity of most diagnostic modalities, and the lack of absolute diagnostic criteria resulting in late patient diagnosis and poor survival rates [8, 9]. Worse still, the majority of patients show symptoms only at an advanced stage of disease [5, 6, 9], and the clinical treatment is closely related to the tumor stage, tumor location, and growth pattern [9]. So, diagnosis requires a high level of suspicion in the appropriate clinical setting and a confirmatory constellation of clinical, laboratory, endoscopic, and radiologic data [9].

In order to improve diagnosis and prognosis, sampling of bile has become a common clinical practice since the introduction of endoscopic retrograde cholangiopancreatography (ERCP) [10, 11]. Exposure of the biliary epithelium to bile and its constituents makes bile an ideal biofluid for analytical profiling studies in malignancy of the biliary tract [12]. The analysis of the cell of bile may therefore provide insights into the pathogenesis of CC, as well as identify biochemical disease markers [13]. It is found that the role of exosomes in the development, diagnosis, and treatment and drug resistance of CC is crucial. Exosomes are 30-100 nm membrane vesicles secreted by a variety of cell types, including tumor cells; they can be generally detected in body fluids such as serum, urine, saliva, bile, ascites, amniotic fluid, and milk [14]. It was developed to utilize bile as a reliable source of miRNA for the potential diagnosis of diseases of the bile duct such as cholangiocarcinoma [15]. So, the change of bile samples of cells for the treatment of patients with CC is important. Analyses of cells of human bile can be done on freshly collected bile samples or different separation solutions. There are many factors that can affect cell vitality in bile including the buffer solution, centrifugal force, centrifugal time, and store time and temperature. In this study, we systematically evaluated four steps in the procedure of bile extraction from patients with CC.

## 2. Materials and Methods

### 2.1. Bile Collection

We collected bile from patients with CC by ERCP (*n* = 10). Approximately 90 mL of bile was obtained from each patient with CC by ERCP, after cannulation of the common bile duct and before contrast injection. We collected a total of 900 mL of bile.

### 2.2. Evaluation of Separating Cell from Patient Bile with Cholangiocarcinoma

**Figure 1:**
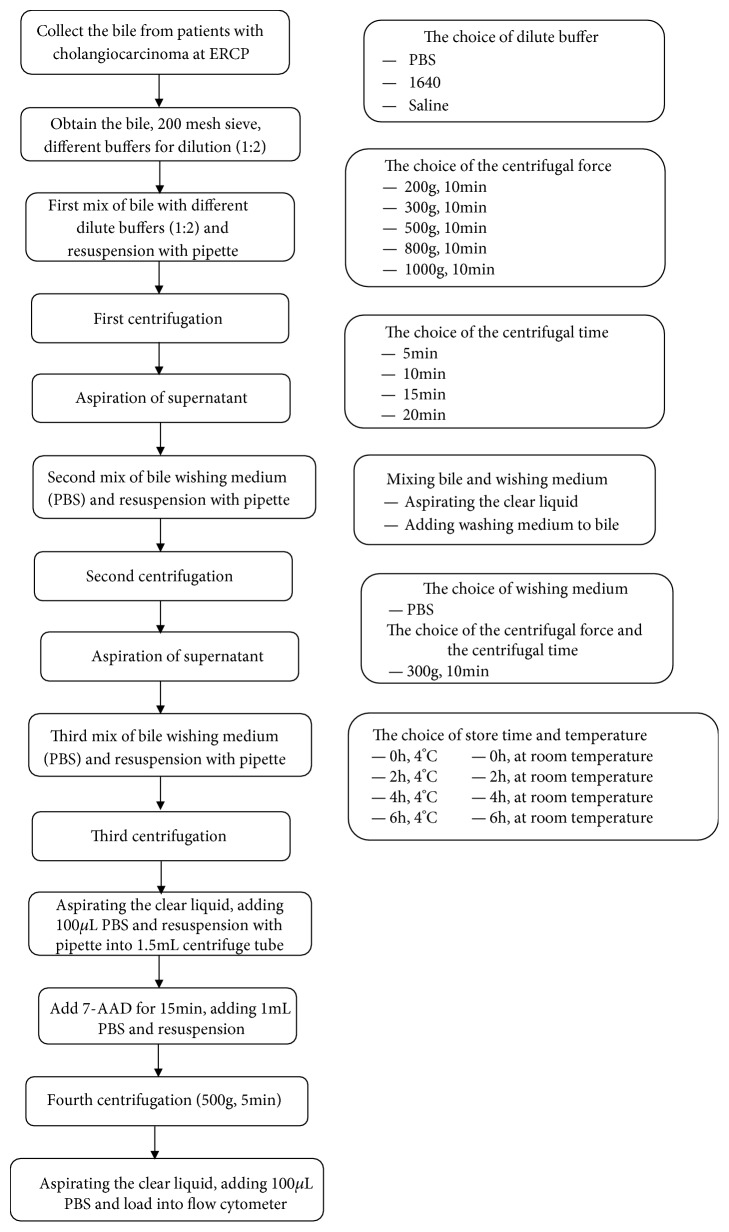
Flow chart of the separation process (on the left) and variations in the separation procedure (on the right).

This experiment explored the key factors that affect cell vitality through the following four steps, including such factors as the dilute buffer, centrifugal force, centrifugal time, and store time and temperature. Flow chart shows the cell separation process from bile and variations in the separation procedure (Figure 1).

### 2.3. Choice of Dilute Buffer

Pour bile samples of about 45 mL into a 50 mL tube, 200-mesh sieve; bile samples are evenly divided into three parts (15 mL), each using three different buffers for dilution (1 : 2); samples are divided into different groups including 1640 culture medium dilution group, PBS dilute group, and the diluted saline group. 45 mL bile sample was filtered through the 200-mesh sieve and evenly put into three centrifuge tubes. Then, different double dilutions were added to bile, including 1640 culture medium, PBS, and saline. The above dilution groups were centrifuged at 300 g for 10 min at 4°C for the formation of the cell layer; then, pour out on the clear liquid after mix in PBS 20 mL, with washing condition at 300 g centrifugation for 10 min at 4°C for the three dilute groups, for the formation of the cell layer, and pour out on the clear liquid after mix in PBS 100 *μ*L. The separated cells from bile were detected by flow cytometry.

### 2.4. Choice of Centrifugal Force and Centrifugal Time

Obtain bile samples of about 135 mL, 200-mesh sieve; 75 mL bile samples are evenly divided into five groups, using different centrifugal forces and the same centrifugal time (10 min), respectively, A (200 g), B (300 g), C (500 g), D (800 g), and E (1000 g). 60 mL bile samples are evenly divided into four groups, respectively, A (5 min), B (10 min), C (15 min), and D (20 min). Each group of 15 mL uses PBS (1 : 2) for dilution. The above groups were centrifuged at different centrifugal forces at 4°C for the formation of the cell layer; then, pour out on the clear liquid after mix in PBS 20 mL with each of the above groups, with washing condition at 300 g centrifugation for 10 min at 4°C for the five groups, for the formation of the cell layer, and pour out on the clear liquid after mix in PBS 100 *μ*L. The separated cells from bile were detected by flow cytometry.

### 2.5. Choice of Store Time and Temperature

Obtain bile samples of about 120 mL, 200-mesh sieve; bile samples are evenly divided into eight groups, using different store times (0, 2, 4, and 6 h) and different temperature (4°C, at room temperature), respectively, group 1 (0 h, 4°C), group 2 (0 h, at room temperature), group 3 (2 h, 4°C), group 4 (2 h, at room temperature), group 5 (4 h, 4°C), group 6 (4 h, at room temperature), group 7 (6 h, 4°C), and group 8 (6 h, at room temperature). Each group of 15 mL uses PBS (1 : 2) for dilution. The above dilution groups were centrifuged at 300 g for 10 min at 4°C for the formation of the cell layer; then, pour out on the clear liquid after mix in PBS 20 mL, with washing condition at 300 g centrifugation for10 min at 4°C for the three dilute groups, for the formation of the cell layer, and pour out on the clear liquid after mix in PBS 100 *μ*L. The separated cells from bile were detected by flow cytometry.

### 2.6. Flow Cytometry

**Figure 2:**
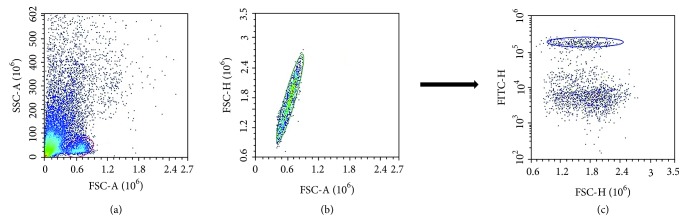
Gating strategy to identify positive cell (CD45). (a) Events were triggered on FSC-H at a deliberately low threshold to avoid accidental exclusion of debris cells. (b) Doublet exclusion. (c) Identification of CD45-positive cells.

After the two washing steps, cells were resuspended in phosphate buffered saline (PBS) and stained with 1 *μ*L FITC-conjugated anti-human-CD45 antibody (BioLegend, clone HI30, catalogue number 304005) for leukocyte count and 1 *μ*L 7-AAD (1 mM) solution (Nordic BioSite, catalogue number ABD-17501) for viability staining. Absolute cell counting was performed using a volumetric NovoCyte flow cytometer (ACEA Biosciences, San Diego, USA). Gating was performed using NovoExpress 1.2.5 software (ACEA Biosciences Inc., San Diego, USA), as visualized in Figure 2.

T-cell population was defined as lineage (CD3, CD4, CD8, CD56, TCR*γ*/*δ*, and CD16). The separated cells from bile were washed and stained with APC-Cy7-conjugated anti-human-CD3 antibody (BioLegend, clone UCHT1, catalogue number 300425), PE-conjugated anti-human-CD4 antibody (BioLegend, clone RPA-T4, catalogue number 300507), PE-Cy7-conjugated anti-human-CD8 antibody (BioLegend, clone HIT8a, catalogue number 300913), FITC-conjugated anti-human-CD56 antibody (BioLegend, clone MEM-188, catalogue number 304603), Percp-conjugated anti-human-TCR*γ*/*δ* antibody (BioLegend, clone B6, catalogue number 331410), and APC-conjugated anti-human-CD16 antibody (BioLegend, clone 3G8, catalogue number 302011). MDSC population was defined as lineage (CD14, HLA-DR, CD33, CD15, CD11b, and Lin). The separated cells from bile were washed and stained with Percp-conjugated anti-human-CD14 antibody (eBioscience, clone 61D3, catalogue number 17-0149-41), FITC-conjugated anti-human-HLA-DR antibody (BioLegend, clone L243, catalogue number 307603), PE-conjugated anti-human-CD33 antibody (eBioscience, clone WM-53, catalogue number 12-0338-41), PE-Cy7-conjugated anti-human-CD15 antibody (BioLegend, clone W6D3, catalogue number 323029), APC-Cy7-conjugated anti-human-CD11b antibody (BioLegend, clone ICRF44, catalogue number 301341), and APC-conjugated anti-human-Lin antibody (BioLegend, clones UCHT1, HCD14, HIB19, 2H7, and HCD56, catalogue number 348703). Neutrophil population was defined as lineage (CD16, CD14, CD15, CD66b, CD11b, and CD45). The separated cells from bile were washed and stained with APC-conjugated anti-human-CD16 antibody (BioLegend, clone 3G8, catalogue number 302011), Percp-conjugated anti-human-CD14 antibody (eBioscience, clone 61D3, catalogue number 17-0149-41), PE-Cy7-conjugated anti-human-CD15 antibody (BioLegend, clone W6D3, catalogue number 323029), PE-conjugated anti-human-CD66b antibody (BioLegend, clone 6/40c, catalogue number 392903), APC-Cy7-conjugated anti-human-CD11b antibody (BioLegend, clone ICRF44, catalogue number 301341), and FITC-conjugated anti-human-CD45 antibody (BioLegend, clone HI30, catalogue number 304005).

**Figure 3:**
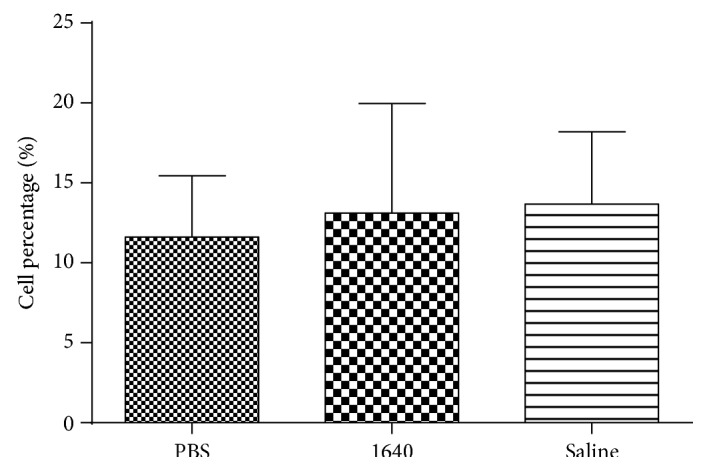
Choice of dilute buffer (PBS, 1640, and saline) by flow cytometry.

**Figure 4:**
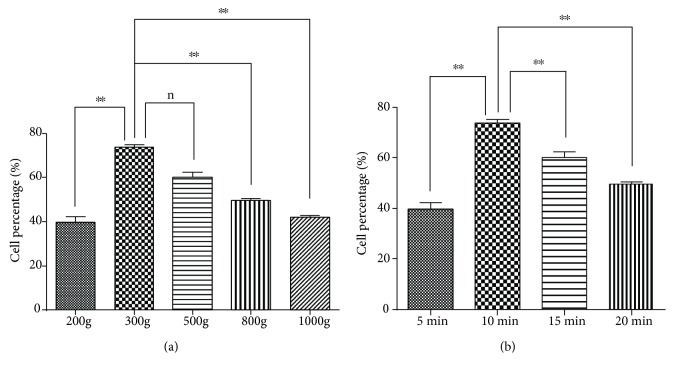
Choice of centrifugal force and centrifugal time by flow cytometry. Impact of centrifugal force on cell percentage (a) and centrifugal time on cell percentage (b). ^∗∗^*p* < 0.01; n: nonsignificant. Choice of centrifugal force is 300 g.

**Figure 5:**
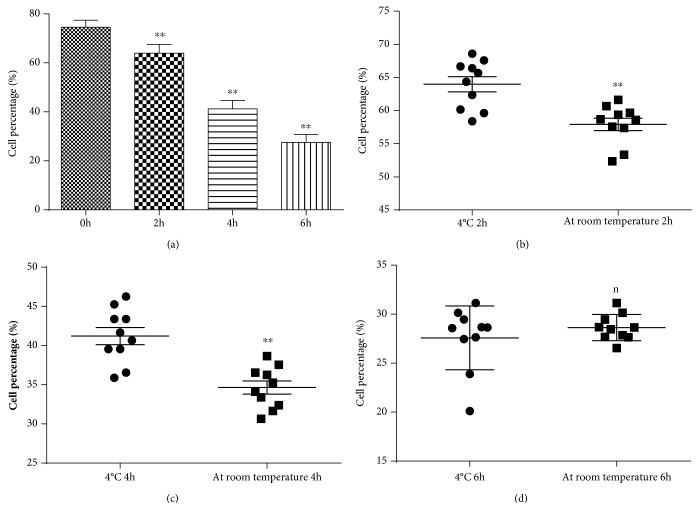
Choice of store time and temperature by flow cytometry. Impact of store time on cell percentage (a), store temperature (4°C, room temperature) for 2 h (b), store temperature (4°C, room temperature) for 4 h (c), store temperature (4°C, room temperature) for 6 h (d). ^∗∗^*p* < 0.01; n: nonsignificant.

Data are presented as mean ± standard deviation (SD) and analyzed by using the statistical analysis software SPSS11.5. We performed a Shapiro-Wilk test on 20 sets of data before applying *t*-test or ANOVA. *p* > 0.05 is considered to be subject to normal distribution. The following is the test result, and all *p* > 0.05. Comparisons were analyzed by one-way ANOVA and were compared using paired *t*-test. *p* values were 2-sided and *p* < 0.01 was considered statistically significant. Figures 3–5 were made using PRISM (GraphPad Software, CA, USA).

## 3. Results

### 3.1. Dilute Buffer

The three different buffer solutions (1 : 2) were joined in bile of patients with CC. The percentage of immune cells has no statistical difference (*p* > 0.05) between the three groups, including the PBS dilute group, the 1640 culture medium dilution group, and the diluted saline group (Figure 3). Therefore, in order to consider the economic factors and convenient conditions, we choose phosphate buffered saline (PBS).

### 3.2. Centrifugal Force

Under the same centrifugal time, we examined the effect of cell viability in bile with different centrifugal forces. For this evaluation, the cell vitality is affected by centrifugal force. The mean viability of centrifugal forces 200 g, 300 g, 500 g, 800 g, and 1000 g was, respectively, 29.54%, 73.69%, 62.16%, 38.19%, and 32.46%. According to statistical results, comparing centrifugal forces 200 g, 800 g, and 1000 g with 300 g, there are significant differences (*p* < 0.01). However, comparing centrifugal force 500 g with 300 g, there is no statistical difference (*p* > 0.05) (Figure 4(a)).

### 3.3. Centrifugal Time

Under the same centrifugal force, we examined the effect of cell viability in bile with different centrifugal times. The mean viability of centrifugal times 5 min, 10 min, 15 min, and 20 min was, respectively, 39.86%, 73.69%, 60.10%, and 49.67%. According to statistical results, comparing centrifugal times 5 min, 15 min, and 20 min with 10 min, there are significant differences (*p* < 0.01) (Figure 4(b)).

### 3.4. Store Time and Temperature

We compared bile stored at 4°C for 0 h, 2 h, 4 h, and 6 h after separation of immune cells from bile and then observe the change of cell vitality by flow cytometry. The mean viability of store times 0 h, 2 h, 4 h, and 6 h was, respectively, 74.55%, 63.97%, 41.22%, and 27.58%. Comparing the store times 2 h, 4 h, and 6 h with 0 h, there are significant differences (*p* < 0.01). So, the shorter the time bile is stored, the stronger the viability of the cells (Figure 5(a)).

We compared bile stored at 4°C and room temperature for 2 h, 4 h, and 6 h after separation of immune cells from bile and then observe the change of cell vitality by flow cytometry (Figures 5(b)–5(d)). Mean viability was higher for cells stored at 4°C for 2 h (63.97%), 4 h (41.22%), and 6 h (27.58%) than at room temperature for 2 h (57.92%, *p* < 0.01), 4 h (34.65%, *p* < 0.01), and 6 h (28.63%, *p* > 0.05). Comparing store temperature at 4°C for 2 h and 4 h with store temperature at room temperature for 2 h, there is significant difference (*p* < 0.01). However, comparing store temperature at 4°C for 6 h with store temperature at room temperature for 6 h, there is no statistical difference (*p* > 0.05).

### 3.5. Immune Cell Subsets Accumulate in Bile from Patient with Cholangiocarcinoma

**Figure 6:**
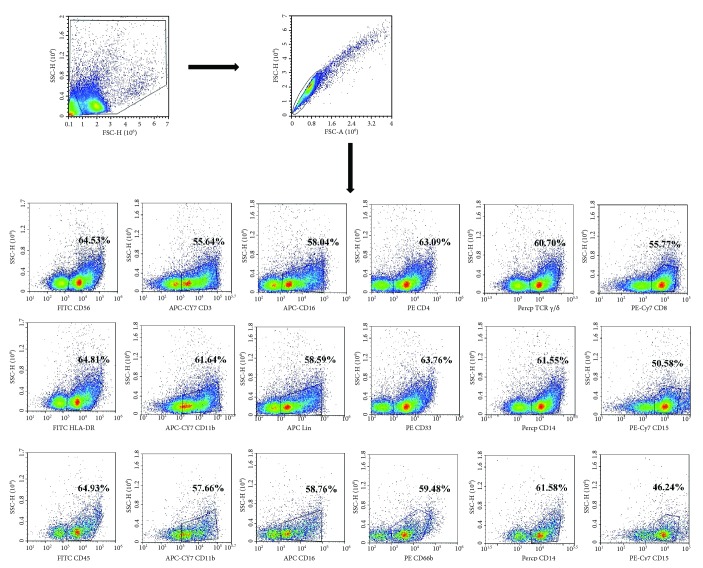
Immune cell subsets accumulate in bile from patient with cholangiocarcinoma by flow cytometry. Proportion of immune cells and distribution of immune cells. The immune cells were stained with FITC-conjugated anti-human-CD56, APC-Cy7-conjugated anti-human-CD3, APC-conjugated anti-human-CD16, PE-conjugated anti-human-CD4, Percp-conjugated anti-human-TCR*γ*/*δ*, and PE-Cy7-conjugated anti-human-CD8 antibody. The cell positive rates are 64.53%, 55.64%, 58.04%, 63.09%, 60.70%, and 55.77%. The immune cells were stained with FITC-conjugated anti-human-HLA-DR, APC-Cy7-conjugated anti-human-CD11b, APC-conjugated anti-human-Lin, PE-conjugated anti-human-CD33, Percp-conjugated anti-human-CD14, and PE-Cy7-conjugated anti-human-CD15 antibody. The cell positive rates are 64.81%, 61.64%, 58.59%, 63.76%, 61.55%, and 50.58%. The immune cells were stained with FITC-conjugated anti-human-CD45, APC-Cy7-conjugated anti-human-CD11b, APC-conjugated anti-human-CD16, PE-conjugated anti-human-CD66b, Percp-conjugated anti-human-CD14, and PE-Cy7-conjugated anti-human-CD15 antibodies. The cell positive rates are 64.93%, 57.66%, 58.76%, 59.48%, 61.58%, and 46.24%.

Expression of T cells, MDSCs and neutrophils in separated cells from bile were marked by antibody. T-cell population was defined as lineage (CD3, CD4, CD8, CD56, TCR*γ*/*δ*, and CD16). The separated cells from bile were washed and stained with anti-human-CD56, CD3, CD16, CD4, TCR*γ*/*δ*, and CD8 antibodies; the cell positive rates are 64.53%, 55.64%, 58.04%, 63.09%, 60.70%, and 55.77% (Figure 6). MDSC population was defined as lineage (CD14, HLA-DR, CD33, CD15, CD11b, and Lin). The separated cells from bile were washed and stained with anti-human-HLA-DR, CD11b, Lin, CD33, CD14, and CD15 antibodies; the cell positive rates are 64.81%, 61.64%, 58.59%, 63.76%, 61.55%, and 50.58% (Figure 6). Neutrophil population was defined as lineage (CD16, CD14, CD15, CD66b, CD11b, and CD45). The separated cells from bile were washed and stained with anti-human-CD45, CD11b, CD16, CD66b, CD14, and CD15 antibodies; the cell positive rates are 64.93%, 57.66%, 58.76%, 59.48%, 61.58%, and 46.24% (Figure 6).

## 4. Discussion

We systematically evaluated four steps in the process of separation by measuring cell viability and absolute immune cells from the bile count. The immune cells from the bile count depended significantly on the choice of dilute buffer, centrifugation force, centrifugation time, and store time and temperature. Within the tested range, we found no effect of changing the dilute buffer, centrifugation time, and force. But storage temperature and storage time significantly influenced bile cell viability. The larger centrifugal force and longer centrifugation time make it easier to obtain cells from bile.

To reliably utilize immune cells isolated from bile, it is important to employ consistent high-quality isolation methods to obtain high-quality samples in return. The methodology defined in this paper is a well-established way to isolate immune cells from human bile. It highlights three steps in the characterization of the isolated cells by flow cytometry. The most crucial step in the isolation of immune cells is to process fresh bile as soon as possible [15].

The diagnosis of occult CC can be very challenging. At present, histological investigation is the standard diagnosis for CCA. However, there are some poorly defined tumor tissues which cannot be definitively diagnosed by general histopathology [16]. Our current research was aimed at identifying biomarkers through bile aspiration which could facilitate the early diagnosis of CC patients [17]. For patients with advanced stage or unresectable cholangiocarcinoma, the available systemic therapies are of limited effectiveness. However, immunotherapy has become a new approach to cancer treatment after surgery, radiotherapy, and chemotherapy in recent years. Among the most promising approaches to activate therapeutic antitumor immunity is the blockade of immune checkpoints [18].

Tumors not only effectively escape immune recognition, but they also actively inhibit T-cell-mediated normal antitumor activity to promote further tumor growth and metastasis by modulating immune checkpoints, which mediate immune tolerance and inhibit the antitumor immune response [19]. Multiple checkpoint molecules, such as PD-1/PD-L1, CTLA4, BTLA, B7H3, B7H4, HHLA2, IDO1, Tim-3, CD28, CD40, CD47, CD70, CD137, VISTA, LAG-3, and TIGIT, have been reported [19]. The clinical activity of antibodies that block either of these receptors implies that antitumor immunity can be enhanced at multiple levels. We focus on the CTLA4 and PD1 pathways because these are the two immune checkpoints for which clinical information is currently available [20, 21]. The FDA approval of anti-CTLA4 therapy and anti-PD1 therapy has engendered a new-found awareness among oncologists of the potential antitumor activity of a patient's endogenous immune system once the “brakes” elicited by the immune system have been released [18, 22].

Immune cells are essential for protection from pathogenic infections and preventing or reducing neoplastic growth [23]. The sophisticated molecular mechanisms can prove how the immune system could combat cancer cells by recognizing cancer cells and killing them [24]. The immune system has different types of immune cells, which have different functions [25, 26]. For example, T cells play an active role in combating cancer cells, while B cells play an innate role in attacking cancer cells. The relationship between immune cells and tumors is evolving with time and cancer progression. Cancer cells bypass immunological suppression and proliferate uncontrollably [27]. Analyses of immune cells from human bile will bring new hope for CC.

## 5. Conclusion

Laboratories worldwide use different approaches when separating immune cells from bile. Here, we show that the freshly collected bile samples have crucial impact on the recovery of live cells. The different buffer solutions were joined in bile of patients with CC; experiment results show that the different dilutions have nearly no effect on the ratio of cells in bile by flow cytometry. The centrifugation force and centrifugation time have a great influence on the vitality of cells. The larger the centrifugation force or the longer the centrifugation time, the more easily cells were separated from the bile. However, the ideal centrifugal force and centrifugation time cannot be met and the cells will not be settled down. Therefore, an optimized method for isolating cells from bile in patients with cholangiocarcinoma is to centrifuge at 300g for 10 min. The shorter the time bile is stored, the stronger the viability of the cells.

## Data Availability

The data used to support the findings of this study are included within the article.
